# Concerted Solvent Processes for Common Sulfonyl Chloride Precursors used in the Synthesis of Sulfonamide-based Drugs

**DOI:** 10.3390/ijms9050914

**Published:** 2008-05-24

**Authors:** Malcolm J. D’Souza, Lamia Yaakoubd, Stacey L. Mlynarski, Dennis N. Kevill

**Affiliations:** 1 Department of Chemistry, Wesley College, 120 N. State Street, Dover, Delaware 19901-3875, USA; E-mail: DSouzaMa@Wesley.edu; 2 Department of Chemistry and Biochemistry, Northern Illinois University, DeKalb, Illinois 60115-2862, USA; E-mail: dkevill@niu.edu

**Keywords:** solvolysis, sulfonyl transfer, 2-thiophenesulfonyl chloride, phenylmethanesulfonyl chloride, Grunwald-Winstein equation

## Abstract

Specific rates of solvolysis in hydroxylic solvents available for the solvolysis of 2-thiophenesulfonyl chloride and phenylmethanesulfonyl chloride are supplemented by determining the values in fluoroalcohol-containing solvents. The data sets are then correlated using the extended Grunwald-Winstein equation. For both substrates, it is found that a single correlation controls the influence of solvent over the full range of solvent composition. The sensitivities to solvent nucleophilicity and solvent ionizing power are compared to values available for other substrates. Three of these previous studies are upgraded by the incorporation of additional specific rate values from the recent literature. With a methyl, isopropyl, benzyl, aromatic or heteroaromatic group as the R group of RSO_2_Cl, a concerted S_N_2 mechanism is proposed for the solvolysis.

## 1. Introduction

The mechanism of solvolysis of sulfonyl halides has been the subject of several reports and reviews. For arenesulfonyl chlorides and alkanesulfonyl chlorides other than tertiary, a bimolecular nucleophilic substitution, believed to be concerted, is usually the dominant pathway [1, 2]. The tertiary alkanesulfonyl chloride, 2-methyl-2-propanesulfonyl chloride, undergoes a solvolysis-decomposition reaction, with extrusion of SO_2_ and formation of *tert*-butyl chloride, 2-methyl propene, and the substitution products expected to be formed *via* the *tert*-butyl cation [2, 3]. In the presence of an α-hydrogen, substitution products can also be formed by an initial elimination reaction to give the sulfene followed by addition of solvent to the highly reactive intermediate. This pathway is especially favored after the addition of a tertiary amine but, with electron-withdrawing substituents also on the α-carbon (trifluoromethyl [4]; chlorine [5], etc.), it can be observed under solvolytic conditions [1, 2, 6].

Sulfonyl chlorides find extensive use in the development of new pharmaceuticals. For example, they have been reacted with a wide variety of amines to give biologically active sulfonamides. Recently, 2-thiophenesulfonyl chloride (**1**) has been found to be a useful reagent in these development projects. A literature search revealed 50 hits for **1** in this area for the year 2007, the majority of them involving patents. Potentially important uses included the development of protein farnesyltranferase (FTase) inhibitors (FTIs) for use as antimalarials [7] and of inhibitors of NS5B polymerase to target the hepatitis C virus [8].

The establishment of a mechanism for the formation of derivatives of **1** by the attack of a nucleophilic species will be helpful in the selection of the optimum reaction conditions. Solvolysis reactions can provide valuable information about the mechanism. One approach [9–12] is to study the influence of solvent variation upon the specific rates of solvolysis, using the extended Grunwald-Winstein equation (equation 1).


(1) $$\text{log k/}k_{0}\text{ = }𝓁\text{N + mY + c}$$

In equation 1, k, and k_0_ are the specific rates (first-order rate coefficients) for solvolysis in a given solvent and in the standard solvent (80% ethanol), respectively, ℓ, represents the sensitivity to changes in solvent nucleophilicity (N_T_), m represents the sensitivity to changes in solvent ionizing power (Y_Cl_, for a chlorideion leaving group), and c is a constant (residual) term.

The specific rates of solvolysis of **1** have been determined previously in several pure and binary solvents [13] but this study did not include any fluoroalcohol-containing solvents. Such solvents are highly recommended for treatments involving equation 1, because the interrelationship of N_T_ and Y_Cl_ is very different to that observed with variation of the composition of binary mixtures of water with conventional alcohols. Prior to carrying out correlations of earlier data using equation 1, we have expanded the range of solvents for which data are available by measuring the specific rates in fluoroalcohol-containing solvents.

**Figure:**
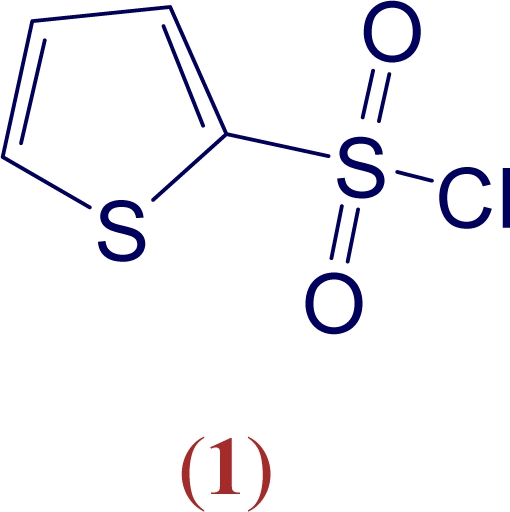


In studies aimed at finding the types of alkanesulfonyl chlorides for which reaction *via* sulfene formation can effectively compete with a direct substitution process, King and Durst [14] studied the solvolysis of phenylmethanesulfonyl chloride (**2**) under both neutral and basic conditions. They found that hydrogen-deuterium exchange *via* PhCD=SO_2_ accompanied the hydrolysis of PhCD_2_SO_2_Cl under basic conditions but not under neutral conditions, where PhCD_2_SO_3_H was the product. We have applied equation 1 to the solvolysis of **2** in a wide variety of solvents to see if any unusual effects are observed for solvolysis of sulfonyl chlorides approaching the border at which direct replacement converts to the indirect elimination-addition replacement route. As with solvolysis of **1**, literature values [15] are supplemented, so as to give a better mix of solvents for the application of equation 1.

## 2. Results and Discussion

**Scheme 1.**
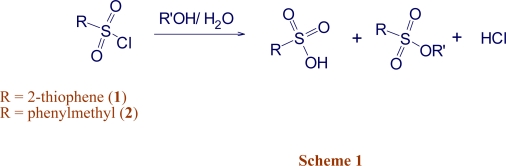


The solvolysis can be represented as in Scheme 1. Under the titration conditions, the sulfonic acid product and the hydrochloric acid are both strong acids towards the sodium methoxide base. For the additional specific rates of solvolysis of 2-thiophenesulfonyl chloride (**1**), three values in aqueous 2,2,2-trifluoroethanol (TFE) and three values in aqueous 1,1,1,3,3,3-hexafluoro-2-propanol (HFIP), together with a value in 80% ethanol were measured at 45.0 °C and, at 25.0 °C, five values in fluoroalcohol containing solvents were obtained and combined with the 29 literature value [15] reported for methanol, ethanol, and binary mixtures of water with methanol, ethanol, and acetone. The new values are reported in Table 1, together with the N_T_ [11] and Y_Cl_ [16–18] values for the solvents. All of the available values at 25.0 °C (34 data points) and 45.0 °C (7 data points) are used to carry out an extended Grunwald-Winstein equation correlation. The ℓ, m, and c values, together with the goodness-of-fit parameters are reported in Table 3. It should be pointed out that seven data points are not really sufficient for a two-term correlation of this type and this is reflected in the low F-test value for this correlation. The reported specific rate for hydrolysis in 100% water of 53.2 × 10^−5^ s^−1^ [15] is in good agreement with an independent value of 47.8 × 10^−5^ s^−1^ [19], indicating the precision involved in the earlier determinations.

Specific rates of solvolysis of **2** were determined at 45.0 °C in 12 fluoroalcohol-containing solvents and in methanol, ethanol, 90% ethanol, and 80% ethanol. These values are reported in Table 2, together with the N_T_ and Y_Cl_ values for the solvents and values for the ratio of the specific rates relative to those previously reported [20, 21] at 45.0 °C for 2-propanesulfonyl chloride (**3**). Values are available [15] at lower temperatures (20–35 °C) for several additional solvents and these have been extrapolated to 45.0 °C using the Arrhenius equation to obtain the following thirteen values for 10^6^ k, s^−1^: 70% EtOH, 204; 60% EtOH, 249; 50% EtOH, 386; 90% MeOH, 149; 80% MeOH, 227; 70% MeOH, 317; 60% MeOH, 382; 50% MeOH, 420; 90% Acetone, 23.1; 80% Acetone, 63.3; 70% Acetone, 92.7; 60% Acetone, 132; 50% Acetone, 195. The 29 specific rate values available (above and in Table 2) have been used in carrying out the correlations reported in Table 3.

Recently, specific rate values have been reported [22] for the solvolyses of 3,4-dimethoxybenzenesulfonyl chloride (**4**) in methanol, ethanol, and several binary water-organic solvent mixtures, including six TFE-water mixtures. Although 42 data points were available, Figure 2 of the publication indicated that only 19 were used in arriving values of 1.12 for ℓ and 0.58 for m. We have carried out the correlation using all 42 specific rate values reported and the results of this correlation are also reported in Table 3.

**Figure:**
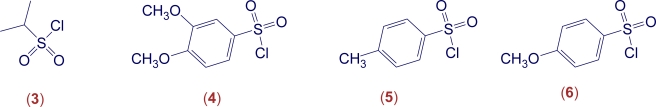


In previous correlations of *p*-methylbenzenesulfonyl chloride (**5**) and *p*-methoxybenzenesulfonyl chloride (**6**) [23], only one TFE-H_2_O data point was available [24] for combination with values in methanol, ethanol, water, and in MeOH-H_2_O, EtOH-H_2_O, and acetone-H_2_O mixtures [25]. Recently specific rate of solvolysis values have become available [26] for both substrates in 97% TFE. Incorporation of the values, as extrapolated to 25.0 °C, considerably improves the data sets and updated sensitivities and goodness-of-fit parameters are reported in Table 3. The presence of six specific rate values in TFE-H_2_O mixtures for the solvolysis of **4**, leads to a considerably improved set of data for use in the correlation. The observation that not only the ℓ and m values but also the goodness-of-fit parameters are extremely similar for the solvolyses of **4**, **5**, and **6** (Table 3) indicates that the analyses of the solvolyses of variously substituted benzenesulfonyl chlorides in terms of equation 1 are very robust.

**Figure 1.**
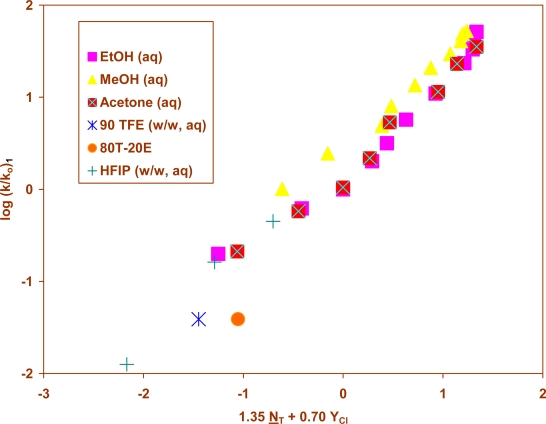
The plot of log (k/k_o_) vs. (1.35 N_T_ + 0.70 Y_Cl_) for the solvolyses of 2-thiophenesulfonyl chloride (**1**) in pure and binary solvents at 25.0 °C.

Correlations of the heteroaromatic sulfonyl chloride (**1**) in 34 solvents at 25.0 °C (Figure 1) led, using equation 1, to a good correlation (R = 0.983; F = 455), with ℓ and m values both slightly higher in value than for substituted benzenesulfonyl chlorides (Table 3). The ℓ/m ratio was, however, essentially identical for the four aromatic sulfonyl chlorides. The ratios, reported in Table 3, are in a narrow range of 1.78 to 2.05. The correlation at 45.0 °C had only seven data points, less than the recommended number for a two-term correlation, but it gave ℓ and m values in reasonable agreement with those from the correlations with considerably more data points, again indicating the correlation to be robust.

An important observation was made by King, Lam, and Skonieczny [27], who found that the specific rates of hydrolysis of a series of alkanesulfonyl chlorides in 100% H_2_O at 25.0 °C varied only from 2.1 × 10^−4^ s^−1^ to 4.4 × 10^−4^ s^−1^ (a factor of two) over a range of R groups in RSO_2_Cl: CH_3_, CH_3_CH_2_, CH_3_CH_2_CH_2_, CH_2_=CH-CH_2_, PhCH_2_, CH_3_OCH_2_CH_2_. In the presence of two alkyl groups on the α-carbon (R = (CH_3_)_2_CH and CH_3_CH_2_CHCH_3_), the specific rates fell to 0.37 × 10^−4^ and 0.35 × 10^−4^, a modest decrease ascribed to a steric effect. As mentioned earlier, when R = (CH_3_)_3_C fragmentation accompanies the solvolysis with substitution and elimination products formed by product-determining reaction of the *tert*-butyl cation [2, 3]. The small rate variation over a wide variety of primary and secondary alkyl groups within the alkanesulfonyl choride suggests that the hydrolyses are proceeding by a mechanism with little charge development at the sulfur; a situation which can best be rationalized in terms of a nearly synchronous process of bond making and breaking.

Since the 2-methyl-2-propanesulfonyl chloride, containing the *tert*-butyl group, proceeds by a solvolysis-decomposition pathway, the same would be expected for other sulfonyl chlorides which can generate a relatively stable carbocation. We have previously shown [20] that 2-propanesulfonyl chloride (**3**), containing the secondary isopropyl group, solvolyzes over the full range of solvents with adherence to equation 1 and with ℓ and m values essentially identical (Table 3) to those for arenesulfonyl chlorides, methanesulfonyl chloride, and N,N-dimethylsulfamoyl chloride. Also the ℓ/m ratio of 2.00 is within the rather narrow range observed for these substrates and the hydrolysis rate, as mentioned above, is only slightly less than for a wide range of alkanesulfonyl chlorides containing a primary alkyl group. One of the primary alkyl groups of the study was phenylmethanesulfonyl chloride (**2**), suggesting that its hydrolysis, as least, proceeds by bimolecular attack at sulfur. This would indeed be not surprising since as a general approximation, it is usually assumed [28] that two methyl groups on the α-carbon (as in **3**) assist the formation of the carbocation to approximately the same extent as one phenyl group (as in **2**). This is supported by the observation that for all the solvents examined in the present study the k (**2**)/k (**3**) specific-rate ratios are in the range of 5–12, except for higher values (20–50) in 70–90% HFIP.

**Figure 2.**
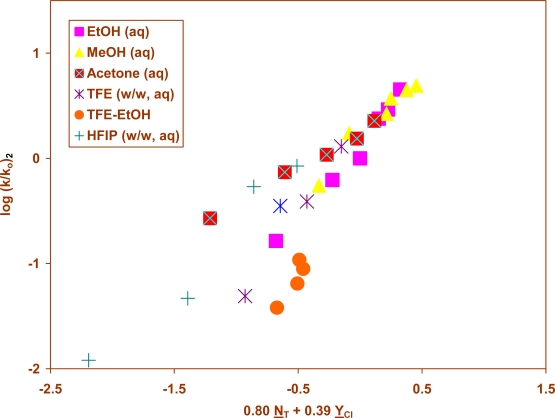
The plot of log (k/k_o_) vs. (0.80 N_T_ + 0.39 Y_Cl_) for the solvolyses of phenylmethanesulfonyl chloride (**2**) in pure and binary solvents at 45.0 °C. The data for TFE-ethanol solvents are not included in the correlation; they are added to show the extent of their deviation from the correlation.

A correlation using equation 1, showed that the modest variations in the specific-rate ratios are accompanied by lower values for both ℓ and m. The points for the TFE-EtOH mixtures on the plots, as is often (but not always) the case [29], fall below the plot and the correlation is improved by their omission from the correlation. Such a correlation (Figure 2), for 25 solvents, leads to values for ℓ and m of 0.80 and 0.39, respectively. The low sensitivity values cannot result from reaction involving solvent attack at the α-hydrogens (sulfene-intermediate mechanism) because it has been shown [27], by deuterium labeling, that hydrogen exchange during hydrolysis occurs only in appreciably basic solution. The ℓ/m ratio of 2.05 is of a typical magnitude for reactions proceeding by S_N_2 attack at sulfur and the sensitivity values are probably best rationalized in terms of the operation of such a process, but with a somewhat earlier transition state. Methanesulfonyl chloride has a ℓ/m ratio of 2.39 [30].

## 3. Conclusions

The specific rates of solvolysis of the heteroaromatic sulfonyl chloride (**1**), when correlated against N_T_ and Y_Cl_ using the extended Grunwald-Winstein equation for all 34 available solvents at 25.0 °C, lead to ℓ and m values which are slightly higher than those obtained for solvolysis of **4** and for two earlier correlations of *para*-substituted benzenesulfonyl chlorides (Table 3). The goodness-of-fit parameters include a multiple correlation coefficient of 0.983 and an F-test value of 455, very high values considering that the N_T_ values, established for attack at carbon, are here being applied to a nucleophilic attack by the solvent at sulfur.

The solvolyses of phenylmethanesulfonyl chloride (**2**) are of interest in that hydrolysis under basic conditions has previously shown to exhibit a dominant pathway involving elimination-addition rather than the direct substitution observed under neutral conditions [27]. Since the benzyl cation is resonance stabilized, one must consider the possibility of a component from the solvolysis-decomposition pathway, which was shown to be dominant for 2-methyl-2-propanesulfonyl chloride hydrolysis [3]. However, consistent with a lack of deuterium exchange under neutral conditions and with the correlation showing typical ℓ and m values over the full range of solvents for solvolyses of 2-propanesulfonyl chloride [20], the specific rates of solvolysis of **2** can be correlated by equation 1 over the full range of solvents, except that the data points for TFE-ethanol solvents fall below the correlation line (Figure 2), as is often the case [29]. The ℓ and m values (0.80 and 0.39, respectively) are low for the correlation of a sulfonyl chloride but the ℓ/m ratio of 2.05 is similar to those for other sulfonyl chlorides, including a value of 1.93 for **1**. A recent report of specific rates of solvolysis for *p-methyl* (**5**) and *p-methoxy-* (**6**) benzenesulfonyl chlorides in 97% TFE [26] allows the correlation in terms of equation 1 to be considerably improved.

It is possible to rationalize the close to “constant” ℓ/m ratios, despite more pronounced variation in the values of ℓ and m themselves, in terms of the variation in the position of the transition state along the reaction coordinate for an S_N_2 nucleophilic substitution at sulfur.

## 4. Experimental Section

The 2-thiophenesulfonyl chloride (Aldrich, 96%) and phenylmethanesulfonyl chloride (Aldrich, α-toluenesulfonyl chloride, 98%) were used as received. Solvents were purified and the kinetic runs carried out as described previously [31]. A substrate concentration of approximately 0.004 M was employed.

The calculation of the specific rates of solvolysis used infinity titers which were calculated using a modified treatment [32] based on the Guggenheim method [33]. For the faster reactions, experimental values for the infinity titer could be obtained and these were in good agreement with the estimated values. The infinity titers were then incorporated into the calculation of a series of integrated rate coefficients for each run. The specific rates and their associated standard deviations, as presented in Tables 1 and 2, are obtained by averaging the values from, at least, duplicate runs.

## Figures and Tables

**Table 1.** First-order rate coefficients (k) for the solvolysis of 2-thiophenesulfonyl chloride (**1**) in binary solvent mixtures of 25.0 and 45.0°C, together with the appropriate solvent nucleophilicity (N_T_) and solvent ionizing power (Y_Cl_) values.

Solvent (%)*a*	10^6^k, s^−1^		N_T_ *b*	Y_Cl_ *c*
	**25.0°C**	**45.0°C**		
80% EtOH		78.7±3.2	0.00	0.00
90% TFE	0.397±0.020	3.88±0.11	−2.55	2.85
80% TFE		21.6±0.7	−2.22	2.90
50% TFE		87.2±8.5	−1.73	3.16
80T-20E*d*	0.986±0.051		−1.76	1.89
90% HFIP	0.129±0.006	2.36±0.17	−3.84	4.31
70% HFIP	1.67±0.10	16.0±0.8	−2.94	3.83
50% HFIP	4.63±0.18	40.6±2.0	−2.49	3.80

a On a weight-weight basis, except for the two ethanol-containing solvents which are on a volume-volume basis at 25.0°C.

b Values from ref. 11.

c Values from refs. 16–18.

d T-E are TFE-ethanol mixtures

**Table 2.** First order rate coefficients (k) for the solvolysis of phenylmethanesulfonyl chloride (**2**) at 45.0°C together with the appropriate solvent nucleophilicity and solvent ionizing power values for the solvents, and a comparison with the corresponding literature values for the solvolyses of 2-propanesulfonyl chloride (**3**).

Solvent (%)*a*	10^6^k, s^−1^	NT*b*	Y_Cl_ *c*	k(2)/k(3)*d*
100% EtOH	14.0±0.9	0.37	−2.52	8.3
90% EtOH	53.3±2.2	0.16	−0.94	6.3
80% EtOH	85.6±0.5	0.00	0.00	6.3
100% MeOH	47.2±2.8	0.17	−1.17	6.5
90% TFE	4.17±0.28	−2.55	2.85	6.1
80% TFE	30.2±1.4	−2.22	2.90	10.1
70% TFE	34.1±2.9	−1.98	2.96	6.7
50% TFE	111±9	−1.73	3.16	8.5
80T-20E*e*	3.24±0.17	−1.76	1.89	11.7
60T-40E*e*	5.54±0.37	−0.94	0.63	5.7
40T-60E*e*	7.69±0.27	−0.34	−0.48	4.8
20T-80E*e*	9.27±0.26	0.08	−1.42	5.1
97% HFIP	1.02±0.09	−5.26	5.17	(48.6)*f*
90% HFIP	4.03±0.29	−3.84	4.31	42.3
70% HFIP	46.1±2.3	−2.94	3.83	21.3
50% HFIP	72.4±2.0	−2.49	3.80	9.6

a TFE-H_2_O and HFIP- H_2_O on a weight-weight basis, other solvents on a volume-volume basis at 25.0°C.

b Values from ref. 11.

c Values from ref. 16–18.

d Values for solvolyses of **3** from ref. 17.

e T-E are TFE-ethanol mixtures.

f Value for **3** only approximate.

**Table 3.** Correlations of the specific rates of solvolytic displacement at the sulfur of alkanesulfonyl and arenesulfonyl chlorides using the extended Grunwald-Winstein equation (equation 1).

Substrate	T°C	n *a*	ℓ *b*	m *b*	c *b*	R *c*	F *d*	ℓ/m
**4**	25.0	40*e*	1.24±0.07	0.64±0.03	0.14±0.06	0.967	264	1.94
	25.0	19*f*	1.12	0.58	0.16			1.93
**6**	25.0	38*g*	1.07±0.08	0.60±0.03	0.22±0.06	0.967	254	1.78
**5**	25.0	34*g*	1.19±0.07	0.61±0.02	0.20±0.05	0.975	305	1.95
**1**	25.0	34	1.35±0.05	0.70±0.02	0.28±0.05	0.983	455	1.93
	45.0	7	1.21±0.15	0.73±0.13	−0.07±0.15	0.978	43	1.66
MeSO_2_Cl	45.0	39*h*	1.17±0.04	0.49±0.02	0.23±0.05	0.981	454	2.39
**3**	45.0	19*i*	1.28±0.05	0.64±0.03	0.18±0.06	0.988	333	2.00
**2**	45.0	29	0.87±0.10	0.46±0.06	0.09±0.09	0.874	42	1.89
	45.0	25*j*	0.80±0.06	0.39±0.04	0.21±0.06	0.947	95	2.05
(CH_3_)_2_NSO_2_Cl	25.0	32*i*	1.20±0.04	0.72±0.03	0.11±0.04	0.985	478	1.67

a Number of data points.

b With associated standard error.

c Multiple correlation coefficient.

d F-test value.

e Specific rate values from ref. 22.

f Reported for the 19 data points of Fig. 2 of ref. 22.

g The solvents used for the correlations previously reported in ref. 23 plus a value for 97% TFE from ref. 26.

h Values from ref. 30.

i Values from ref. 20.

j Value for TFE-ethanol (T-E) solvents omitted.
